# Identification of a Ubiquitination-Related Gene Risk Model for Predicting Survival in Patients With Pancreatic Cancer

**DOI:** 10.3389/fgene.2020.612196

**Published:** 2020-12-22

**Authors:** Hao Zuo, Luojun Chen, Na Li, Qibin Song

**Affiliations:** ^1^Cancer Center, Renmin Hospital of Wuhan University, Wuhan, China; ^2^Hubei Provincial Research Center for Precision Medicine of Cancer, Wuhan, China

**Keywords:** pancreatic cancer, bioinformatics, prognosis, ubiquitination-related genes, risk model

## Abstract

Pancreatic cancer is known as “the king of cancer,” and ubiquitination/deubiquitination-related genes are key contributors to its development. Our study aimed to identify ubiquitination/deubiquitination-related genes associated with the prognosis of pancreatic cancer patients by the bioinformatics method and then construct a risk model. In this study, the gene expression profiles and clinical data of pancreatic cancer patients were downloaded from The Cancer Genome Atlas (TCGA) database and the Genotype-tissue Expression (GTEx) database. Ubiquitination/deubiquitination-related genes were obtained from the gene set enrichment analysis (GSEA). Univariate Cox regression analysis was used to identify differentially expressed ubiquitination-related genes selected from GSEA which were associated with the prognosis of pancreatic cancer patients. Using multivariate Cox regression analysis, we detected eight optimal ubiquitination-related genes (RNF7, NPEPPS, NCCRP1, BRCA1, TRIM37, RNF25, CDC27, and UBE2H) and then used them to construct a risk model to predict the prognosis of pancreatic cancer patients. Finally, the eight risk genes were validated by the Human Protein Atlas (HPA) database, the results showed that the protein expression level of the eight genes was generally consistent with those at the transcriptional level. Our findings suggest the risk model constructed from these eight ubiquitination-related genes can accurately and reliably predict the prognosis of pancreatic cancer patients. These eight genes have the potential to be further studied as new biomarkers or therapeutic targets for pancreatic cancer.

## Introduction

Pancreatic cancer is a highly fatal disease, with 43,090 deaths every 5 years (Siegel et al., 2017), the 5-year overall survival rate is only 6% (Miller et al., 2019). Many factors contribute to low survival rates for pancreatic cancer. The most important factor may be that more than half of patients are diagnosed with advanced pancreatic cancer, and the 5-year survival rate of advanced pancreatic cancer is only 3% (Ilic and Ilic, 2016). Pancreatic cancer is characterized not only by early recurrence and invasion but also by chemical and radiation resistance (Adamska et al., 2018). In recent years, targeted therapy and emerging immunotherapy have opened up new prospects for the treatment of pancreatic cancer. However, the exploration of new therapeutic targets and prognostic biomarkers for pancreatic cancer still needs to be further carried out. Over the past decade, numerous studies have identified some sensitive and effective biomarkers for pancreatic cancer.

Ubiquitination/deubiquitination is an ATP-dependent reversible reaction that binds small ubiquitin molecules to the target protein through multi-step reactions involving ubiquitin-activating enzyme E1, ubiquitin-binding enzyme E2, and ubiquitin-ligase E3 (Hershko et al., 1979). ATP provides energy, E1 is activated, and the Glycine residue at the Carboxy terminal of ubiquitin and the active Cystine of E1 forms a thioester bond. Next, E1 transfers the ubiquitin to the cysteine residue of the ubiquitin carrier protein E2. E3 is specific in that it coordinates ubiquitin covalently to specific target proteins. The way ubiquitin molecules bind plays an important role in the function of the modified protein (Dikic et al., 2009). Ubiquitination produces a protein that is either monoubiquitinated or polyubiquitinated when one of the seven Lysine residues of ubiquitin binds to the C-terminal Glycine of another ubiquitin. The reverse process of ubiquitination is called deubiquitination. Ubiquitination is best known for its role in mediating protein degradation. Besides, ubiquitination is also involved in the processes of meiosis, autophagy, DNA repair, immune response, and apoptosis. Ubiquitinated proteasome pathway is involved in almost all intracellular molecular biological processes, affecting gene expression and signal transduction in the regulation of DNA damage repair, participating in the differentiation of senescent cells, regulating tumor progression of malignant transformation, and mediating therapeutic resistance (Welchman et al., 2005).

Previous studies have shown that ubiquitination/deubiquitination play important roles in pancreatic cancer. Lian et al. (2020) found that ubiquitin specific peptidase 5 (USP5) enhances STAT3 signaling and promotes migration and invasion in pancreatic cancer. Chen et al. (2020) found that E3 ubiquitin ligase UBR5 promotes pancreatic cancer growth and aerobic glycolysis by downregulating FBP1 via destabilization of C/EBPα. Yang et al. (2019) found that USP44 suppresses pancreatic cancer progression and overcomes gemcitabine resistance by deubiquitinating FBP1. There is no doubt that ubiquitination/deubiquitination is closely related to the progression of pancreatic cancer. Exploration of ubiquitination/deubiquitination related genes in pancreatic cancer is also necessary.

In this study, by analyzing the dataset from TCGA and GTEx database, we aim to study and verify the expression characteristics of ubiquitination-related genes. We then selected several ubiquitination-related genes that were significantly associated with the prognosis of pancreatic cancer patients through a series of statistical methods. Finally, we established a new and reliable risk model to predict the prognosis of pancreatic cancer patients based on the screened risk genes.

## Materials and Methods

### Databases

To download the transcriptome data of 178 patients (The Cancer Genome Atlas database, TCGA database) with pancreatic cancer and the transcriptome data of 36 cases of normal pancreatic tissue (Genotype-Tissue Expression database, GTEx database) from the UCSC XENA website^1^. Clinical information of pancreatic cancer patients was obtained from the TCGA database. All data are processed using R software^2^. The clinical features of pancreatic cancer patients, include age, gender, pathological grade, T-stage, N-stage, M-stage, and TNM-stage.

### Gene Set Enrichment Analysis

GSEA^3^ was used to explore whether the transcriptome data showed statistically significant difference between the two groups (normal and tumor). The expression data of mRNAs, including 36 normal pancreatic tissue and 178 pancreatic cancer samples were analyzed. Normalized P value (*P* < 0.05) and normalized enrichment score (NES) were used to determine what functions had to be selected for further analysis.

### Screening for Differentially Expressed Genes (DEGs)

We screened DEGs from these ubiquitination/deubiquitination related genes obtained from GSEA analysis. The “limma” package was used to screen out the DEGs (Log_2_ fold change ≠ 0, *P* < 0.05).

### GO Analysis and KEGG Analysis

Gene Ontology (GO) database is a kind of free and open database, the database includes three aspects of information: biological process, cellular component, and molecular function. The biological functions of genes can be classified and these genes included in the functions that we selected can be further understood through the GO analysis. DAVID online tool^4^ was used for GO analysis (Xia et al., 2015). Kyoto Encyclopedia of Genes and Genomes (KEGG) database is a database that systematically analyzes the metabolic pathways of gene products in cells and the functions of these gene products. The database is useful for studying gene and expression information as a whole network. KEGG integrates the data of genomic chemical molecules and biochemical systems, including the sequence and genome of metabolic pathways, drugs, and diseases. We used the “clusterProfiler” package (Yu et al., 2012) from Bioconductor to do KEGG analysis of these DEGs. *P*-value < 0.05 was used as the inclusion standard in the analysis.

### Identification and Inclusion of Prognostic Ubiquitination-Related Genes for the Construction of a Risk Model

As in previous studies (Li et al., 2020), univariate Cox regression (*p* < 0.05) was used to screen out the ubiquitination-related genes that were significantly associated with the prognosis of pancreatic cancer patients from the DRGs. Multivariate Cox proportional hazards regression analysis (with forwarding selection and backward selection) was then used to analyze these ubiquitination-related genes selected by univariate Cox regression. Finally, optimal ubiquitination-related genes (risk genes) were obtained to be incorporated into the risk model. The alteration of these risk genes is shown online^5^.

### Construction of the Prognostic Risk Model in Pancreatic Cancer Cohort

Multivariate Cox proportional hazards regression analysis was used to select the optimal risk genes and construct the Cox regression model. In this process, we can obtain the estimated regression coefficients of each gene. The expression levels of mRNA and estimated regression coefficients of the risk genes were used to calculate a risk score for each pancreatic cancer patients. The risk score model was established with the following formula: Risk score = expression level of Gene1 ^∗^ β1 + expression level of Gene2 ^∗^ β2+…+ expression level of Gene_n_^∗^ β_n_; where β is the estimated regression coefficient calculated by the multivariate Cox regression model.

The risk model was used to measure the prognostic risk for each pancreatic cancer patient. The median risk score was used as the cut-off value to divide all the pancreatic cancer patients into two groups: the high-risk group and the low-risk group. The low-risk group has a better prognosis.

### Independent Prognostic Value of the Risk Model in the Pancreatic Cancer Cohort

Next, univariate and multivariate Cox regression analysis were performed to assess whether the risk model was independent of other clinical features (age, gender, pathological grade, T-stage, and N-stage) as a prognostic factor for pancreatic cancer patients (*p* < 0.05). The X-tile software was used to identify the optimal cut-off value of the age significantly correlated to the prognosis of pancreatic cancer patients. Because there are too many patients in M0-stage and too few patients in stage III/IV, we excluded these two clinical features (M-stage and TNM-stage) from this analysis. Besides, cases with incomplete clinical information were also excluded. Then, we constructed receiver operating curves (ROC) and calculated the area under the curve (AUC) to assess whether our model accurately predicted the overall survival (OS) of pancreatic cancer patients. C-index value of 0.75 or greater were considered to have excellent predictive value, and value of 0.6 or greater were considered acceptable for survival predictions (Cho et al., 2019).

### Validation of the Eight-mRNA Model in Predicting Survival Using Kaplan–Meier Curves

Kaplan–Meier curves and the log-rank test were used to validate the prognostic significance of the risk model (*p* < 0.05).

### Validation of the Risk Genes in Protein Level

Furthermore, the Human Protein Atlas database^6^ was used to validate the protein expression level of these risk genes compared to the level of gene transcription.

## Results

### Gene Set Enrichment Analysis

**FIGURE 1:**
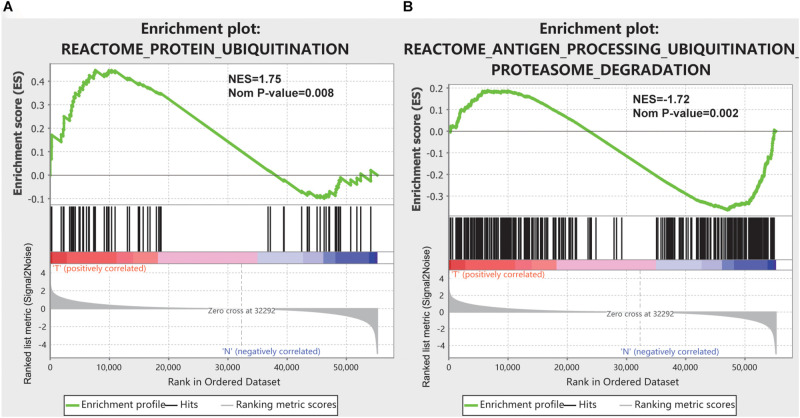
GSEA results for the enrichment plots of two gene sets (REACTOME_ANTIGEN_PROCESSING_UBIQUITINATION_PROTEASOME_DEGRADATION, and REACTOME_PROTEIN_UBIQUITINATION) that were significantly differentiated in normal and pancreatic cancer tissues based on TCGA database. **(A)** Enrichment plot of the REACTOME_ANTIGEN_PROCESSING_UBIQUITINATION_PROTEASOME_DEGRADATION gene set. **(B)** Enrichment plot of the REACTOME_PROTEIN_UBIQUITINATION gene set.

Expression data set for 55242 mRNAs from the TCGA database and GTEx database were analyzed. Five ubiquitination/deubiquitination-related gene sets we validated by GSEA analysis and there were two gene sets, including REACTOME_ANTIGEN_PROCESSING_UBIQUITINATION_ PROTEASOME_DEGRADATION, and REACTOME_ PROTEIN_UBIQUITINATION were significantly enriched (Table 1 and Figure 1). These 441 ubiquitination-related genes in the two functions were selected for the subsequent analysis.

**TABLE 1:** Gene sets enriched in pancreatic cancer.

**Gene sets enriched in pancreatic cancer**		
**GS follow link to MSigDB**	**NES**	**NOM *p*-value**
GO_UBIQUITIN_DEPENDENT_ERAD_PATHWAY	−1.46	0.075
KEGG_UBIQUITIN_MEDIATED_PROTEOLYSIS	−1.45	0.067
REACTOME_ANTIGEN_PROCESSING_UBIQUITINATION_ PROTEASOME_DEGRADATION	−1.72	0.002
REACTOME_DEUBIQUITINATION	1.38	0.085
REACTOME_PROTEIN_UBIQUITINATION	1.75	0.008

### GO Analysis and KEGG Analysis

Of these 441 ubiquitination-related genes in the two functions, 134 DEGs were screened. These 134 ubiquitination-related DEGs were used to do the GO analysis and KEGG analysis. The results of the GO analysis showed that the functions of the ubiquitination-related genes were concentrated in the functions of the protein polyubiquitination, post-translational protein modification, and proteasome-mediated ubiquitin-dependent protein catabolic process, as shown in Table 2. The results of KEGG analysis showed that the functions of the ubiquitination-related genes were concentrated in ubiquitin-mediated proteolysis, proteasome, and cell cycle, as shown in Table 2.

**TABLE 2:** Result of GO and KEGG analysis for these ubiquitination-related DEGs.

**ID**	**Description**	***P*-adjust**	***Q*-value**
**GO analysis**			
GO:0000209	Protein polyubiquitination	<0.001	<0.001
GO:0043687	Post-translational protein modification	<0.001	<0.001
GO:0043161	Proteasome-mediated ubiquitin-dependent protein catabolic process	<0.001	<0.001
GO:0010498	Proteasomal protein catabolic process	<0.001	<0.001
GO:0031145	Anaphase-promoting complex-dependent catabolic process	<0.001	<0.001
GO:0006513	Protein monoubiquitination	<0.001	<0.001
GO:0031146	SCF-dependent proteasomal ubiquitin-dependent protein catabolic process	<0.001	<0.001
**KEGG analysis**			
hsa04120	Ubiquitin mediated proteolysis	<0.001	<0.001
hsa03050	Proteasome	<0.001	<0.002
hsa04110	Cell cycle	<0.001	<0.003
hsa04141	Protein processing in endoplasmic reticulum	<0.001	<0.004
hsa04114	Oocyte meiosis	0.017	0.016
hsa05017	Spinocerebellar ataxia	0.018	0.016
hsa04144	Endocytosis	0.020	0.018
hsa05169	Epstein-Barr virus infection	0.023	0.020
hsa04115	p53 signaling pathway	0.043	0.038

### Identification and Inclusion of Prognostic Ubiquitination-Related Genes for the Construction of a Risk Model

**FIGURE 2:**
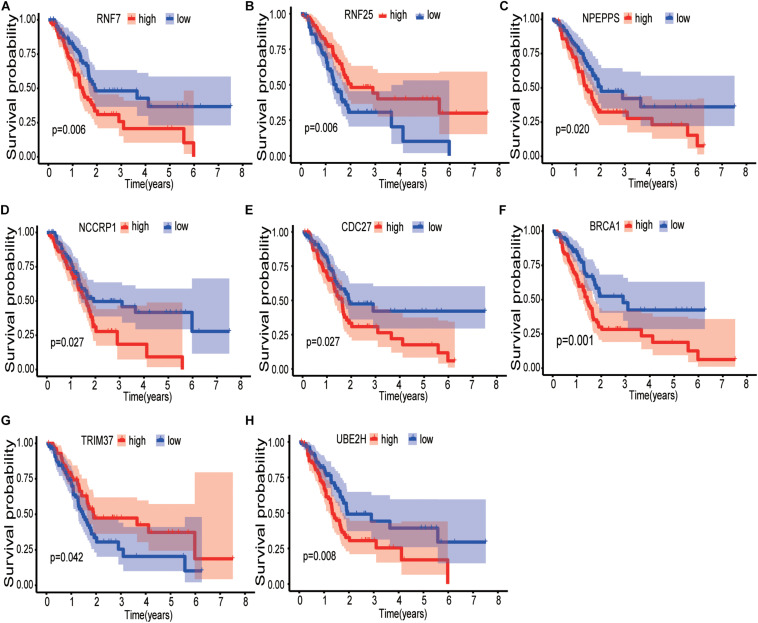
Kaplan-Meier curves of the effect of the gene expression level of the risk genes (RNF7, NPEPPS, NCCRP1, BRCA1, TRIM37, RNF25, CDC27, and UBE2H) on the prognosis of pancreatic cancer patients. **(A)** Kaplan-Meier curve of the effect of RNF7 gene expression level. **(B)** Kaplan-Meier curve of the effect of RNF25 gene expression level. **(C)** Kaplan-Meier curve of the effect of NPEPPS gene expression level. **(D)** Kaplan-Meier curve of the effect of NCCRP1 gene expression level. **(E)** Kaplan-Meier curve of the effect of CDC27 gene expression level. **(F)** Kaplan-Meier curve of the effect of BRCA1 gene expression level. **(G)** Kaplan-Meier curve of the effect of TRIM37 gene expression level. **(H)** Kaplan-Meier curve of the effect of UBE2H gene expression level.

**FIGURE 3:**
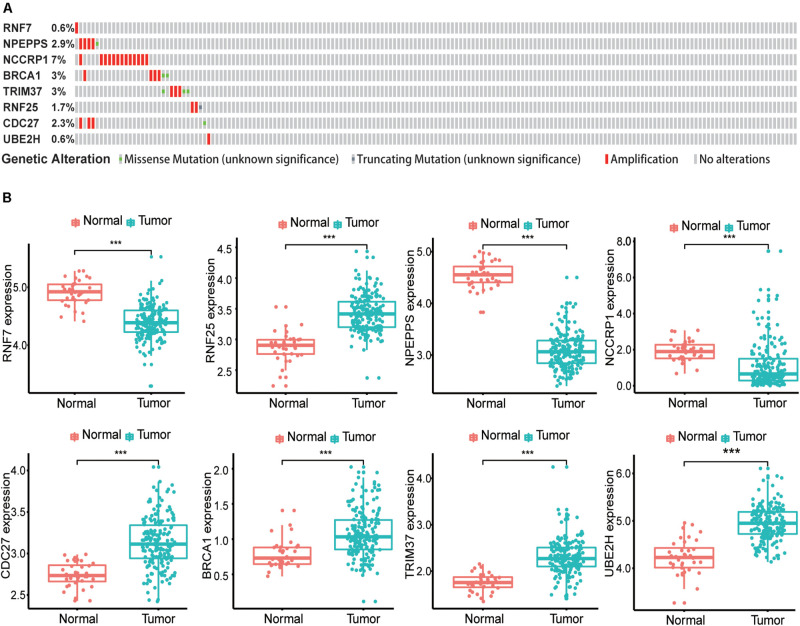
Identification of mRNAs associated with patient survival. **(A)** The alteration proportion for the eight selected genes in 175 clinical samples of pancreatic cancer in the cBioPortal database. **(B)** Different expression of eight genes in the normal pancreatic tissues and tumor tissues based on TCGA database. (*P < 0.05, **P < 0.01, ***P < 0.001).

Sixty-three ubiquitination-related genes significantly correlated with the prognosis of pancreatic cancer patients were screened through the univariate Cox regression analysis from the 134 DEGs. Next, eight optimal ubiquitination-related genes (risk gene) obtained by multivariate Cox analysis were used to construct a risk model (Table 3): RNF7, NPEPPS, NCCRP1, BRCA1, TRIM37, RNF25, CDC27, and UBE2H. The effect of the expression value of these genes on the prognosis of pancreatic cancer is shown in Figures 2A–H. Then, the alteration of the seven genes in 175 clinical pancreatic cancer samples was analyzed in the cBioPortal database. Results showed that there were 33(19%) sequenced cases among the 175 pancreatic cancer samples with the eight genes altering. The alterations of the eight genes are shown in Figure 3A. We also investigated the different expressions of the eight genes between pancreatic cancer tissues and normal pancreatic tissues. Among the eight genes, five genes (BRCA1, TRIM37, RNF25, CDC27, and UBE2H) were significantly upregulated and three genes (RNF7, NPEPPS, and NCCRP1) were significantly down regulated in the tumor tissues (Figure 3B).

**TABLE 3:** The detailed information of eight prognostic mRNAs significantly associated with the prognosis of pancreatic cancer patients.

**The detailed information of eight prognostic mRNAs significantly associated with the prognosis of pancreatic cancer patients**						
**mRNA**	**Official name**	**Ensemble ID**	**Location**	**β (Cox)**	**HR (95% CI)**	***p*-value**
RNF7	Ring finger protein 7	ENSG00000114125	Chr3: 141, 738, 209-141, 747, 560	2.3538	10.526 (3.759, 29.475)	<0.001
NPEPPS	Aminopeptidase puromycin sensitive	ENSG00000141279	Chr17: 47, 522, 933-47, 623, 276	–1.0029	0.367 (0.154, 0.875)	0.024
NCCRP1	NCCRP1, F-box associated domain containing	ENSG00000188505	Chr19: 39, 196, 964-39, 201, 884	0.2271	1.255 (1.061, 1.484)	0.008
BRCA1	BRCA1 DNA repair associated	ENSG00000012048	Chr17: 43, 044, 295-43, 125, 364	1.1898	3.286 (1.543, 7.001)	0.002
TRIM37	Tripartite motif containing 37	ENSG00000108395	Chr17: 58, 968, 010-59, 106, 880	–1.6370	0.195 (0.081, 0.469)	<0.001
RNF25	Ring finger protein 25	ENSG00000163481	Chr2: 218, 663, 874-218, 672, 002	–1.5668	0.209(0.087, 0.502)	<0.001
CDC27	Cell division cycle 27	ENSG00000004897	Chr17: 47, 117, 703-47, 189, 295	1.9902	7.317 (2.672, 20.037)	<0.001
UBE2H	Ubiquitin conjugating enzyme E2 H	ENSG00000186591	Chr7: 129, 830, 732-129, 952, 960	1.0606	2.888 (1.392, 5.991)	0.004

### Construction of the Prognostic Risk Model in Pancreatic Cancer Cohort

Finally, 171 pancreatic cancer patients were included in the risk model. The computational formula was as follows: Risk score = (2.3538 × expression of RNF7) + (−1.0029 × expression of NPEPPS) + (0.2271 × expression of NCCRP1) + (1.1898 × expression of BRCA1) + (−1.6370 × expression of TRIM37) + (−1.5668 × expression of RNF25) + (1.9902 × expression of CDC27) + (1.0606 × expression of UBE2H).

**FIGURE 4:**
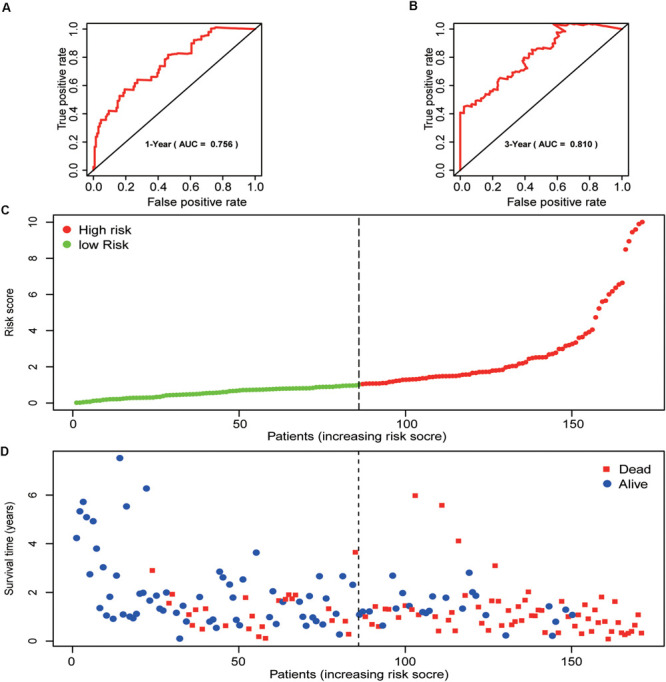
Prognostic analysis of the pancreatic cancer cohort. **(A)** 1-year ROC curve analysis of the prognostic model. **(B)** 3-year ROC curve analysis of the prognostic model. **(C)** Risk score distribution of patients in the prognostic model. **(D)** Survival status scatter plots for patients in the prognostic model.

Patients were divided into two groups, the high-risk group (*n* = 85) and the low-risk group (*n* = 86). The high-risk group had a worse outcome than the low-risk group (*p* < 0.001). The 1- and 3-year OS of pancreatic cancer patients in the high-risk group were 87.7 and 64.7%, respectively, while the corresponding OS in the low-risk group was 57.5 and 17.9%, respectively. The AUC (ROC) value of the risk model in 1-year, and 3-year were 0.756, and 0.810, respectively (Figures 4A,B). Then, risk scores of these pancreatic cancer patients were ranked and their distribution was analyzed. We divided pancreatic cancer patients into low-risk and high-risk groups by the median risk score for all patients enrolled in the study (Figure 4C). The survival status of each patient in the pancreatic cancer patients was shown in Figure 4D. As can be intuitively seen from Figure 4D, the higher the risk score, the shorter the OS of pancreatic cancer patients.

### Independent Prognostic Value of the Risk Model in the Entire Pancreatic Cancer Cohort

A total of 163 pancreatic cancer patients were included in this analysis. Results of the univariate analysis showed that age, pathological grade, T-stage, N-stage, and risk score were significantly correlated with the prognosis of pancreatic cancer patients. The result of multivariate analysis showed that the risk score was independently correlated with the OS for patients with pancreatic cancer (Table 4).

**TABLE 4:** Effects of various clinical features on pancreatic cancer patients.

		**Univariate analysis**			**Multivariate analysis**		
**Clinical feature**	**Number**	**HR**	**95% CI**	***p*-value**	**HR**	**95% CI**	***p*-value**
Age (<62/62–76/>76)	58/83/22	1.028	1.005–1.052	0.016	1.550	0.919–2.614	0.100
Gender (female/male)	75/88	0.768	0.499–1.183	0.232	0.825	0.530–1.285	0.394
Grade (G1/2/G34)	114/49	1.387	1.001–1.924	0.049	1.243	0.777–1.990	0.364
N-stage (N0/N1)	45/118	2.004	0.999–4.021	0.050	1.229	0.589–2.563	0.583
T-stage (T1/2/T3/4)	25/138	2.222	1.286–3.838	0.004	1.598	0.890–2.869	0.116
Risk score (low/high)	86/85	1.284	1.207–1.367	<0.001	1.250	1.172–1.333	<0.001

### Validation of the Eight-mRNA Signature in Predicting Survival Using Kaplan–Meier Curves

**FIGURE 5:**
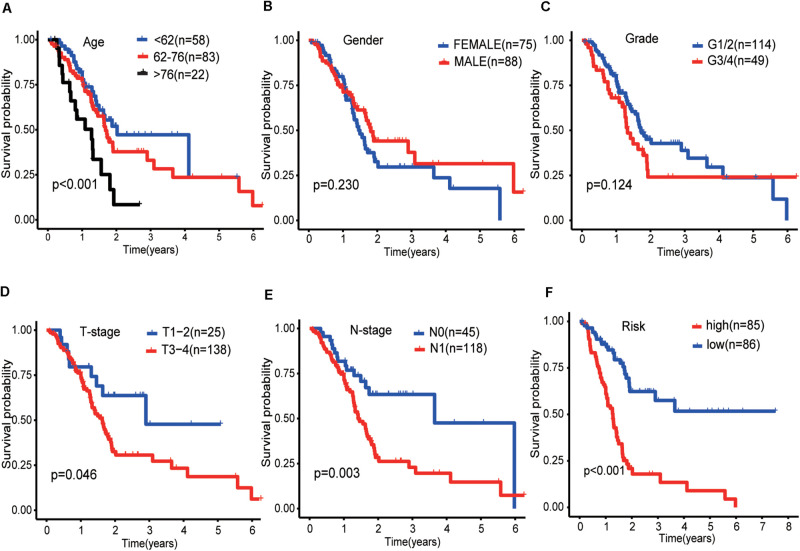
Kaplan-Meier curves of the effect of clinical features (risk score, age, gender, pathological grade, T-stage, and N-stage) on the prognosis of pancreatic cancer. **(A)** Kaplan-Meier curve of the effect of age. **(B)** Kaplan-Meier curve of the effect of gender. **(C)** Kaplan-Meier curve of the effect of pathological grade. **(D)** Kaplan-Meier curve of the effect of T-stage. **(E)** Kaplan-Meier curve of the effect of N-stage. **(F)** Kaplan-Meier curve of the effect of risk score.

**FIGURE 6:**
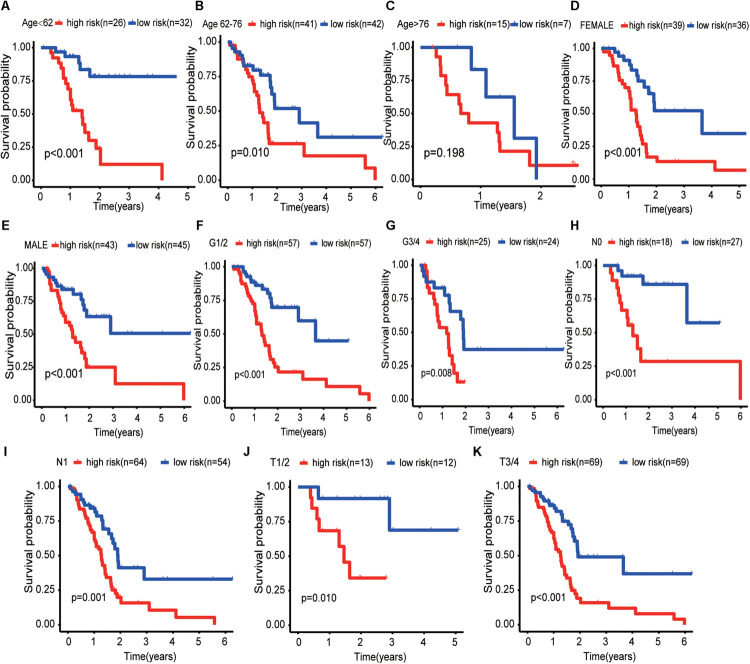
Kaplan–Meier curves for the prognostic value of risk model for the patients grouped according to each clinical feature. **(A–C)** Kaplan–Meier survival curves of the age patient group (<62, 62–76, and >76). **(D,E)** Kaplan–Meier survival curves of the gender patient group (male and female). **(F,G)** Kaplan–Meier survival curves of the pathological grade patient group (G1/2 and G3/4). **(H,I)** Kaplan–Meier survival curves of the T-stage patient group (T1/2 and T3/4). **(J,K)** Kaplan–Meier survival curves of the N-stage patient group (N0 and N1).

The results of the univariate analysis showed that age was an independent prognostic factor for pancreatic cancer, and the X-tile software found that 62 and 76 were the optimal cut-off values for the prognosis of pancreatic cancer patients (Supplementary Figure 1). The result of Kaplan–Meier curves showed the effects of age, gender, histological grade, T-stage, N-stage, and risk score on the prognosis of pancreatic cancer patients (Figures 5A–F). The result of Kaplan–Meier curves showed that our risk model was a stable predictive tool for the prognosis of pancreatic cancer patients stratified by age (<62, 62–76, and >76), gender (male and female), pathological grade (G1/2, or G3/4), T-stage (T1/2, or T3/4), and N-stage (N0 or N1) (Figures 6A–K). Patients with pancreatic cancer in the high-risk group had significantly shorter OS than those in the low-risk group when the patients were stratified into different subgroups based on age, gender, pathological grade, T-stage, and N-stage.

### Validation of the Risk Genes

**FIGURE 7:**
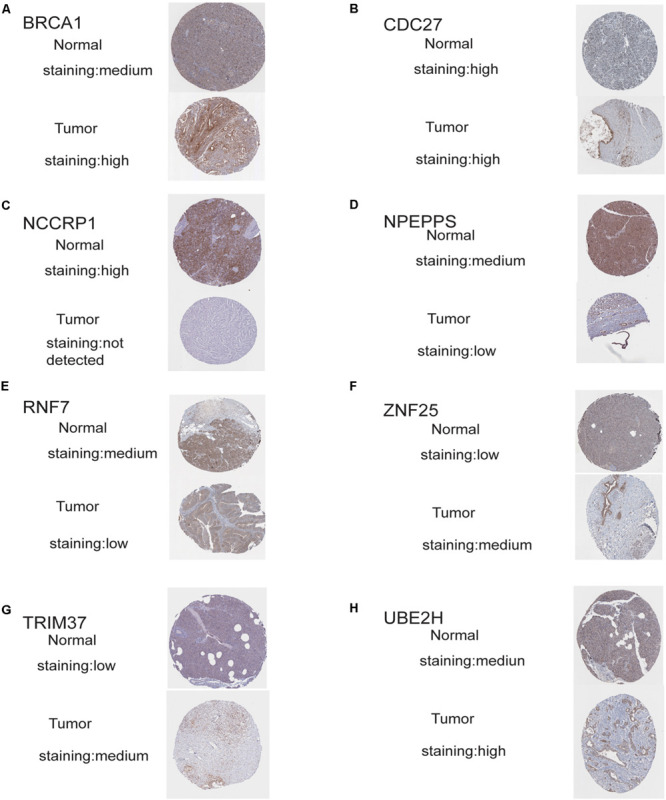
Validation of risk genes at the translational level. **(A)** Validation of BRCA1 by The Human Protein Atlas database (IHC). **(B)** Validation of CDC27 by The Human Protein Atlas database (IHC). **(C)** Validation of NCCRP1 by The Human Protein Atlas database (IHC). **(D)** Validation of NPEPPS by The Human Protein Atlas database (IHC). **(E)** Validation of ZNF7 by The Human Protein Atlas database (IHC). **(F)** Validation of ZNF25 by The Human Protein Atlas database (IHC). **(G)** Validation of TRIM37 by The Human Protein Atlas database (IHC). **(H)** Validation of UBE2H by The Human Protein Atlas database (IHC).

The protein levels of immunohistochemistry (IHC) staining obtained from the HPA database showed that the expression of the protein in four risk genes (BRCA1, TRIM37, RNF25, and UBE2H) was significantly higher in pancreatic cancer tissues than in normal pancreatic tissues, three genes (RNF7, NPEPPS, and NCCRP1) do the opposite, which was consistent with that at the transcriptional level. Only CDC27 protein expression levels was high in both the normal and tumor group in the HPA database (Figures 7A–H).

## Discussion

One or more pathway data sets are used to assess the ranking list of statistically significant genes/proteins using GSEA. GSEA can not only detect statistically significant genes and proteins group-wise but also enrich the previous research characteristics of gene sets in functional genomes in a large database of pathway gene sets (Subramanian et al., 2005; Wu et al., 2014). In our study, mRNA expression data from 178 patients with pancreatic cancer and 36 normal pancreatic tissues were used for GSEA analysis, and significant differences were found in two functions. These two functions are all related to ubiquitination, indicating that ubiquitination changes significantly in the development of pancreatic cancer. And then, these ubiquitination-related genes in the two functions were selected for subsequent analysis.

Combined with GO enrichment analysis and KEGG enrichment analysis, the results suggest that these genes are closely related to the ubiquitination process of pancreatic cancer. Next, eight optimal ubiquitination-related genes were identified via multivariate Cox proportional hazards regression analysis, and they were used to construct a risk model. The reliability and stability of the model were further validated. The results showed that the model could accurately distinguish pancreatic cancer patients with different survival outcomes. The results of univariate and multivariate analysis showed that our model could independently predict the outcome of pancreatic cancer patients. The result of Kaplan–Meier curves shows that our risk model has excellent stability and reliability in predicting the prognosis of pancreatic cancer at all age, gender, pathological grade, T-stage, and N-stage. Therefore, our risk model can screen high-risk patients for personalized treatment. Finally, the eight risk genes were validated by the HPA database, and the results showed that the protein expression level of the eight genes was generally consistent with those at the transcriptional level. These results suggest that the genes we identified deserve further study.

Of the eight genes identified, seven genes (RNF7, NCCRP1, BRCA1, TRIM37, RNF25, CDC27, and UBE2H) have been reported to play roles in ubiquitination (Asamitsu et al., 2003; Kallio et al., 2011; Link et al., 2016; Cho et al., 2018; Lim and Joo, 2020; Meitinger et al., 2020; Zhang et al., 2020). It has not been reported that NPEPPS directly participates in the process of ubiquitination, but NPEPPS is also known to degrade the tau protein, which accumulates and polymerizes in some neurodegenerative diseases (Kudo et al., 2011). In our study, the expression of these ubiquitination-related genes was significantly associated with the prognosis of patients with pancreatic cancer, providing us with a new key to the study of pancreatic cancer. Among these genes, some have been studied as biomarkers for cancer. For example, BRCA has been proved to be a biomarker in many cancers, and its mutation or not has a guiding role in the application of targeted drugs, such as pancreatic cancer (Wu and Shi, 2020). RNF7, an apoptosis-sensitive gene, has been shown in several previous studies to play an important role in the development and progression of tumors such as prostate cancer and lung cancer (Li et al., 2014; Tan et al., 2016). There are also relevant studies showing that RNF7 regulates ionizing radiation-induced apoptosis in pancreatic cancer (Kim et al., 2011). TRIM37 has also been shown to promote the proliferation, invasion and migration in breast cancer, lung cancer, gastric cancer, glioma, and pancreatic cancer (Jiang et al., 2016; Li et al., 2018; Tang et al., 2018; Hu et al., 2019; Fu et al., 2020). CDC27 promotes the progression and affects PD-L1 expression in T-cell lymphoblastic lymphoma, and also promotes epithelial-to-mesenchymal transition in colorectal cancer (Qiu et al., 2017; Song et al., 2020). There are few studies on the role of NCCRP1, RNF25, and UBE2H in cancer, but the existing research results suggest that these three genes also have the potential to become new tumor biomarkers or targets for cancer (Miwa et al., 2017; Cho et al., 2018; Zhu et al., 2018).

Of the eight genes we identified, three genes (RNF7, NPEPPS, and NCCRP1) were down-regulated and the remaining five (BRCA1, TRIM37, RNF25, CDC27, and UBE2H) were up-regulated in tumor tissue compared to normal pancreatic tissue. But we found that even though some genes (RNF7, NPEPPS, and NCCRP1) were down-regulated in tumor group, patients with pancreatic cancer with high expression of these genes had a worse prognosis. Some genes are up-regulated (TRIM37 and RNF25), but high expression of these genes has a better prognosis. So we suspect that these genes play an opposite role in the development and progression of pancreatic cancer. For example, NPEPPS may inhibit tumor formation in normal tissue but may promote tumor progression once the tumor has formed. This phenomenon has been reported in previous literature. In retrospect, the study has shown that TGF-β is a key negative regulator of cell proliferation, but the abnormal function of retinoblastoma protein can lead to the inhibition of the function of TGF-β and promote the progression of pancreatic cancer (Gore et al., 2014). Another study showed that Daple is also a tumor-suppressor gene, although it appears only in the early stages of cancer to function as a tumor-suppressor gene. In the later stages of cancer, when cancer cells escape from their primary sites and circulate in the blood, the expression of Daple makes cancer cells more aggressive and more likely to spread (Aznar et al., 2015).

Many previous studies have explored new potential biomarkers and therapeutic targets for pancreatic cancer through bioinformatic methods. Wu et al. (2019) screened nine DEGs (MET, KLK10, COL17A1, CEP55, ANKRD22, ITGB6, ARNTL2, MCOLN3, and SLC25A45) through the joint analysis of GEO and TCGA databases and construct a risk score model. They also analyzed the relationship between the nine gene models and tumor immune infiltration. Wei et al. (2019) constructed a risk model to predict the prognosis of pancreatic cancer patients by screening nine immune-related lnRNAs from the TCGA database. Compared with the previous studies, we use GSEA enrichment analysis to explore the function of ubiquitination in pancreatic cancer, and on this basis, identify eight ubiquitination-related genes to construct a risk model. There has been no previous study on the bioinformatics related to the ubiquitination of pancreatic cancer, and our study provides a new idea for relevant studies on the progression of pancreatic cancer.

Of course, our study also has some shortcomings. First, our study was a retrospective study based on a public database. The data we used has not been validated by prospective clinical trials. Besides, the identified mechanism of ubiquitination-related genes affecting the development of pancreatic cancer needs further support from basic experimental studies. Next, we need to collect clinical specimens and data for subsequent studies.

## Conclusion

Using GSEA enrichment analysis, we found that the ubiquitination-related functions of pancreatic cancer were significantly different from those of normal pancreatic tissues. Subsequently, we extracted and screened the genes in these functions, and finally selected eight genes significantly related to the prognosis of pancreatic cancer patients as risk genes to construct a risk model. This model has a good predictive effect on the prognosis of pancreatic cancer patients. Moreover, these eight genes have the potential to be further studied as new biomarkers or therapeutic targets for pancreatic cancer.

## Data Availability Statement

The datasets supporting the conclusions of this article are obtained from The Cancer Genome Atlas (TCGA) portal website (https://portal.gdc.cancer.gov/) and the Genotype-Tissue Expression (GTEx) database (https://xenabrowser.net/). The alteration of these genes is from an online database (http://www.cbioportal.org/). The authors did not have special access privileges.

## Author Contributions

NL and QS: conception and design. HZ and LC: data acquisition, data analysis and interpretation, and article writing and revision. All authors have read and agreed to the published version of the manuscript.

## Conflict of Interest

The authors declare that the research was conducted in the absence of any commercial or financial relationships that could be construed as a potential conflict of interest.

## Footnotes

1 https://xenabrowser.net/

2 https://www.r-project.org/

3 http://www.broadinstitute.org/gsea/index.jsp

4 http://david.ncifcrf.gov/

5 http://www.cbioportal.org/

6 https://www.proteinatlas.org/

## Supplementary Material

Supplementary Figure 1 The result of the best cut-off values for the age by using the X-tile software.
