# Supplementary figures and images for: Identification of a Ubiquitination-Related Gene Risk Model for Predicting Survival in Patients With Pancreatic Cancer
Source: Front Genet. 2020 Dec 22;11:612196. doi: 10.3389/fgene.2020.612196 (PMC7782244; doi:10.3389/fgene.2020.612196)

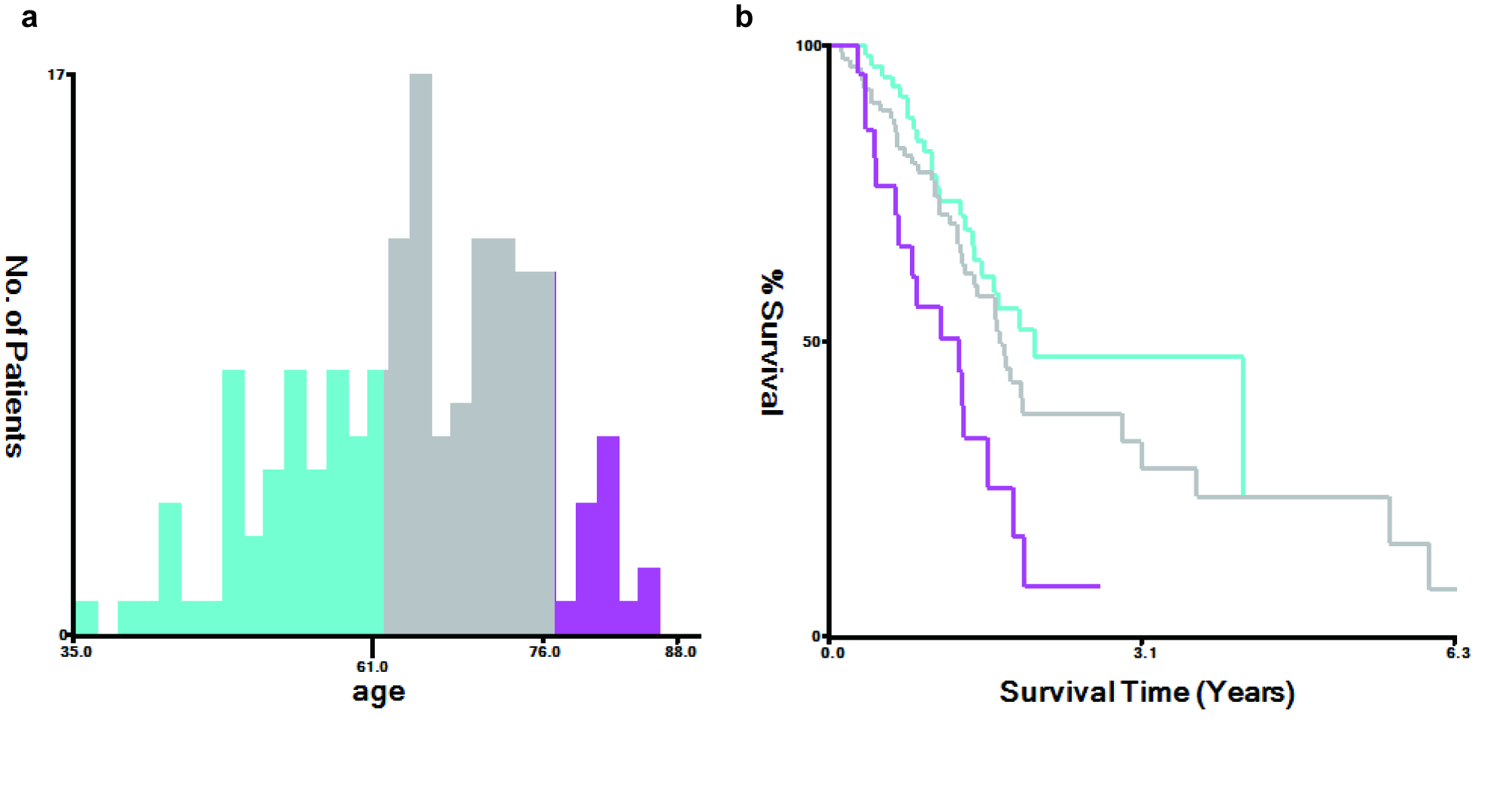

Supplement: Supplementary Figure 1 — The result of the best cut-off values for the age by using the X-tile software. [file Image_1.TIF]
